# Highly efficient organic tandem solar cell with a SubPc interlayer based on TAPC:C_70_ bulk heterojunction

**DOI:** 10.1038/srep23916

**Published:** 2016-04-01

**Authors:** Yuan Gao, Fangming Jin, Wenlian Li, Zisheng Su, Bei Chu, Junbo Wang, Haifeng Zhao, Hairuo Wu, Chengyuan Liu, Fuhua Hou, Tong Lin, Qiaogang Song

**Affiliations:** 1 State Key Laboratory of Luminescence and Applications, Changchun Institute of Optics, Fine Mechanics, and Physics, Chinese Academy of Sciences, Changchun 130033, People’s Republic of China; 2 University of Chinese Academy of Sciences, Beijing 100039, People’s Republic of China

## Abstract

We report a small molecule tandem organic photovoltaic (OPV) cell with a high power conversion efficiency (PCE) of 7.27%. This cell contains two subcells with an identical mixed active layer of C_70_:5 wt%TAPC (1,1-bis-(4-bis(4-methyl-phenyl)-amino-phenyl)-cyclohexane). The performance was dramatically improved by simply inserting a thin boron subphthalocyanine chloride (SubPc) interlayer, which results in an increase of the short-circuit current and open-circuit voltage as well as a decrease of the series resistance of the tandem cell. The response of the cell only contributed from the absorption of C_70_. The high PCE was attributed to the high absorption efficiency of C_70_ and improved holes extraction efficiency at the anode due to the band bending occurs at both MoO_3_/SubPc and SubPc/C_70_:5 wt%TAPC interfaces.

Over the past few years, organic photovoltaic (OPV) cells have drawn a great deal of attention in both fundamental research and industrial fields. OPV cell is one of the most-promising next-generation energy-harvesting technologies because it has the advantages of low weight, flexible form factor, low-cost mass production and low energy consumption in manufacturing processes^1^,^2^,^3^,^4^,^5^,^6^,^7^,^8^. Recently, the power conversion efficiency (PCE) of the OPV cells has exceeded 10%, which is a milestone development for valuable commercial applications^9^,^10^. In spite of this, there are some factors limit the performance of OPV cells such as low light absorption and charge carrier collection efficiencies. A tandem cell architecture is usually adopted to increase the absorption and hence the performance of OPV cells. Besides, the architecture of tandem cells should be precisely designed to guarantee a high charge carrier collection efficiency. Typically, tandem cells are constructed by series stacking two or more subcells with complementary absorption spectra because the overall light absorption could be enhanced. As a result of such a design, a matched photocurrent resulted from the individual subcell was extracted^11^. The subcells with identical compositions have also been used in tandem structure, but the PCE of these cells was generally lower^12^,^13^. Zhang *et al*.^13^ reported a tandem cell with the same subcell units of MoO_3_/C_60_:5 wt% 1,1-bis-(4-bis(4-methyl-phenyl)-amino-phenyl)-cyclohexane (TAPC). This subcell structure was similar to their previous reported OPV cells^14^, in which the open circuit voltage (Voc) was mainly determined by the MoO_3_/C_60_:TAPC Schottky barrier^15^ and the short circuit current (Jsc) was primary originated from the absorption of the fullerene. This tandem OPV cells had a lower PCE of 4.12%. This should be attributed the low absorption efficiency of C_60_ and low charge carrier collection efficiency of this device^13^. In one of our previous works, we have observed a considerable improved PCE of the OPV cell when a thin boron subphthalocyanine chloride (SubPc) interlayer was inserted between ITO/MoO_3_ and bulk heterojunction (BHJ) of TAPC:C_60 _16. A high hole extraction efficiency has been obtained in planar heterojunction (PHJ) OPV cells with SubPc and fullerene materials as the donor and acceptor, respectively^17^,^18^. Because SubPc has a deep highest occupied molecular orbital (HOMO) level (i.e., higher ionization potential), a significant band bending towards the gap states in the MoO_3_ layer^18^ due to interface dipole effect can be observed at the MoO_3_/SubPc interface^17^,^19^. Such an energy level alignment allows more efficient holes extraction of the devices^17^. C_70_ fullerene has comparable electronic properties but a higher extinction coefficient at low energy wavelength region as compared with C_60_ fullerene. This leads to a higher PCE of OPV cells with a C_70_ acceptor.

In this work, tandem OPV cells with consistent subcell that comprising a C_70_:5 wt%TAPC active layer is designed. By inserting a thin SubPc layer between MoO_3_ and the TAPC:C_70_ BHJ layer, a high PCE of 7.27% is obtained. In light of the above descriptions, our new tandem cell system exist two MoO_3_-SubPc Schottky junctions which lead to a stronger built-in field in the cell due to a thinner SubPc and a BHJ active layer were adopted in the subcells. As a result of such a structure, we can expect a high charge carrier extraction efficiency and hence a high PCE of the tandem cell. Our optimized tandem cell was addressed by series stacking the front- and back-cells, which has an architecture of ITO/MoO_3_ (5 nm)/SubPc (1 nm)/5 wt%TAPC:C_70_ (33 nm)/Bphen (2 nm)/Ag (0.5 nm)/MoO_3_ (5 nm)/SubPc (1 nm)/5 wt%TAPC:C_70_ (54 nm)/Bphen (8 nm)/Al (100 nm). In the end, a peak PCE of 7.27% was successfully demonstrated, which is one of the highest value among the reported tandem OPV cells with two identical subcells. It is also interesting that the increase in spectral response of our new single cell mainly focuses on the absorption of C_70_ acceptor. That is, there was no complementary absorption in our new tandem cell, which is in contrast to conventional reported tandem cells^20^. Besides, the donor and acceptor materials used here are all conventional and extensive materials. Therefore, we believe that cost-efficient OPV cells can be developed thanks to the design of the tandem cell architecture and the utilization of facile materials, for example, C_70_, SubPc, TAPC, Ag, and so on.

## Results and Discussion

**Figure 1:**
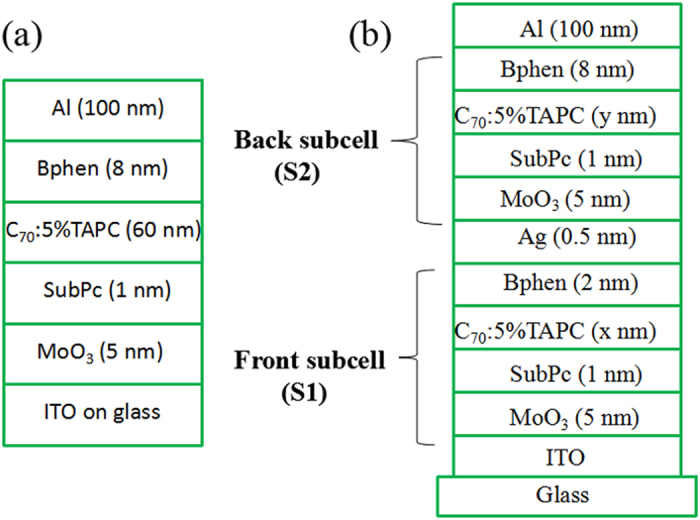
Device architecture of the single cell and (**b**) tandem cell with changed thickness ratio between front and back subcell.

**Figure 2:**
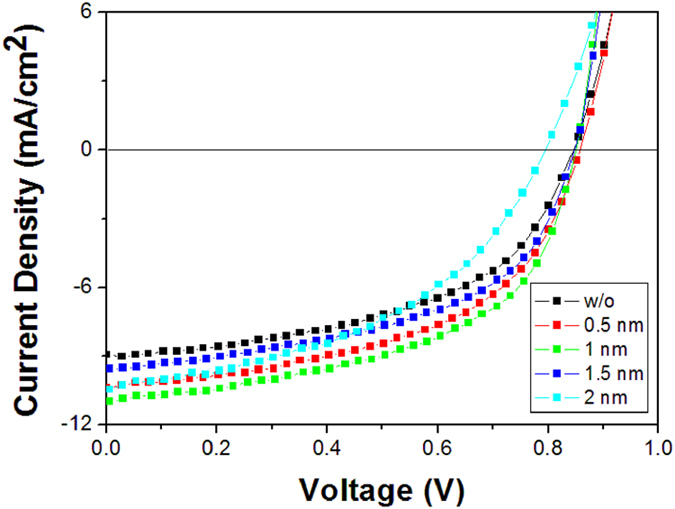
The J–V curves of single cells with different thickness of SubPc.

Prior to constructing the tandem OPV cells, a series of single cells with the structure of ITO/MoO_3_ (5 nm)/SubPc (x nm)/C_70_:5 wt%TAPC (60 nm)/Bphen (8 nm)/Al (100 nm) were constructed, here x ranged from 0.5 to 2.0 nm, as shown in Fig. 1(a). The J–V curves of these cells are presented in Fig. 2, and the PV parameters extracted from these curves are listed in Table 1. Each curve is averaged over four devices with the same configuration. The reference cell without SubPc layer shows a Jsc, Voc, fill factor (FF), and PCE of 8.94 mA/cm^2^, 0.85 V, 0.51, and 3.88%, respectively. After inserting a thin SubPc layer, the performance of the cells is significantly improved. The optimized cell with 1 nm SubPc shows a Jsc, Voc, FF, and PCE of 10.98 mA/cm^2^, 0.85 V, 0.53, and 4.95%, respectively. The PCE is increased by 27.6% compared with the reference cell which is mainly ascribed to the increased Jsc and FF. Meanwhile, a decrease of series resistance is also observed (see Table 1), which implies that there is a low carrier recombination probability in these cells.

**Figure 3:**
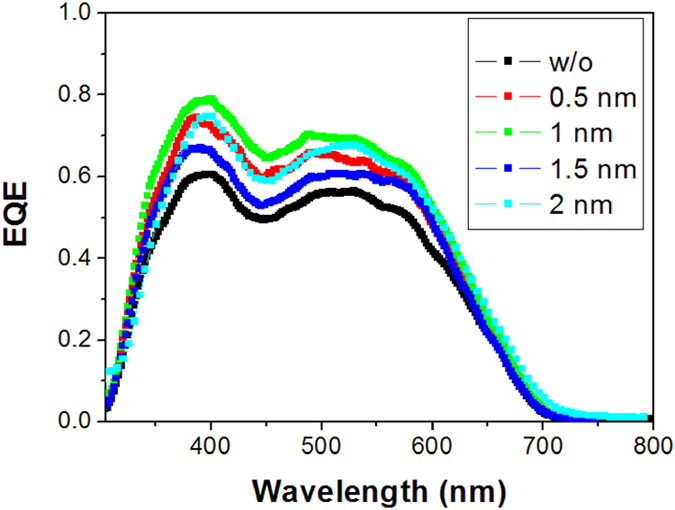
The external quantum efficiency (EQE) spectra of single cells with different thickness of SubPc films.

**Figure 4:**
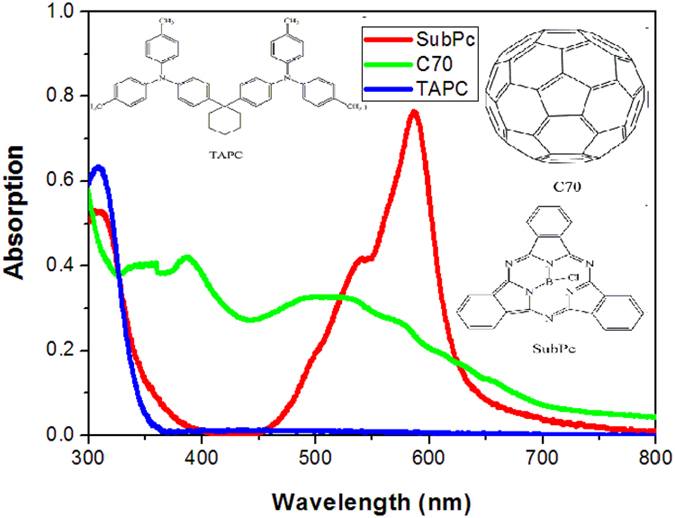
The normalized absorption spectra of the SubPc, C_70_ and TAPC films with 50 nm thickness as well as their chemical structures.

The EQE spectra of the single OPV cells with different thickness of SubPc are showed in Fig. 3. All these cells show a similar shape of EQE spectra with two bands located at about 400 and 550 nm, respectively. Besides, we can find that the cell with a 1 nm SubPc exhibits the highest EQE in the whole visible region, which is consistent with the highest Jsc observed in the J–V curves. The calculated Jsc from this EQE spectrum is 10.71 mA/cm^2^, which is a little lower than that obtained from the J–V curve but still within the range of allowable error. The normalized absorption spectra of 50 nm SubPc, C_70_, and TAPC films are depicted in Fig. 4, as well as their chemical structures. C_70_ has two absorption bands at about 390 and 520 nm, while the absorption band of SubPc locates at about 590 nm. The EQE spectra are more consistent to the absorption spectrum of C_70_, indicating that the photocurrent is predominantly contributed from the absorption of C_70_. To further distinguish the contributions of the absorptions of SubPc and C_70_ to the photocurrent, the absorption spectra of SubPc/C_70_:TAPC films with different SubPc thickness are investigated, as shown in Supplementary Fig. S1. The absorption intensity at about 590 nm which comes from SubPc slightly increases with the thickness of SubPc. However, no obvious respond at 590 nm is found in the EQE spectra regardless of its thickness. The J–V curves and the response of the devices ITO/MoO_3_/SubPc/C_70_/Bphen/Al are shown in Supplementary Fig. S2. The response of the devices exhibit strong peaks at about 590 nm, which can be assigned to the absorption of SubPc. These findings suggest that SubPc does not act as a donor but more likely only as an interlayer in these cells.

Based on these results, we fabricated a series of tandem cells with the structures of ITO/[MoO_3_ (5 nm)/SubPc (1 nm)/C_70_:5 wt%TAPC (x nm)/Bphen (2 nm)](S1)/Ag (0.5 nm)/[MoO_3_ (5 nm)/SubPc (1 nm)/C_70_:5 wt%TAPC (y nm)/Bphen (8 nm)](S2)/Al (100 nm), as depicted in Fig 1(b), here x and y represents the thickness of the active layer, respectively. We can see that the two subcells (S1 and S2) are connected by a 0.5 nm Ag layer which acts as the recombination centre of electrons and holes that originated from S1 and S2, respectively. The transmittance spectra of 0.5 nm Ag and Bphen (2 nm)/Ag (0.5 nm)/MoO_3_ (5 nm) are showed in Supplementary Fig. S3. It can be seen that the transmittances are more than 94% in the range from 300 to 800 nm, ensuring enough light to be absorbed by the top cell. Hence, this intermediate layer satisfies the optical requirement for device fabricated.

**Figure 5:**
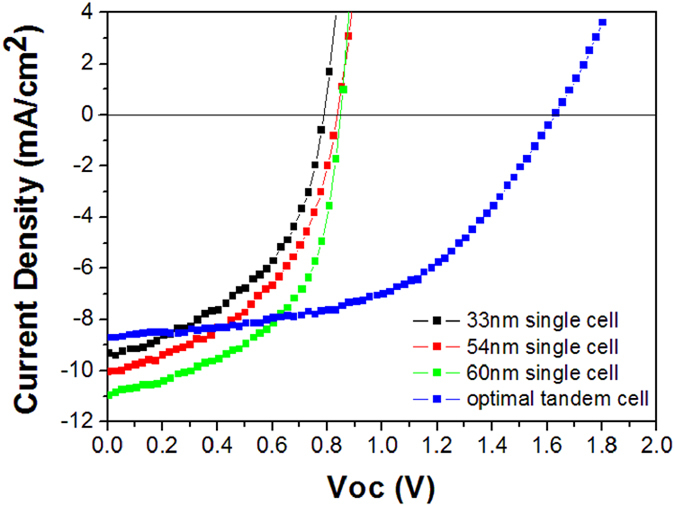
The relation between short curve current density and voltage of the optimized tandem and the corresponding reference single cell with different thickness of active layer.

The PV parameters of the tandem cells with different thickness of front and back subcells are shown in Table 2. Figure 5 compares the J–V curves of the optimized tandem cell and reference single cells. The single cells with 33, 54, and 60 nm C_70_:5 wt% TAPC layers shows a PCE of 3.49, 4.02, and 4.95, respectively, while the optimized tandem cell with 33 and 54 nm C_70_:5 wt% TAPC in S1 and S2, respectively has a maximum PCE of 7.27%. This suggests that the PCE of the tandem cell is about 47% higher than that of the reference single one. We also note that the Voc of the tandem cell is almost double that of the single cell, which indicates that our designed tandem OPV cells are in accordance with the requirements of the tandem OPV cells to improve their performance^13^,^21^,^22^. It is surprising that the FF is almost unchanged for the tandem and single cells due to their lower series resistance, as shown in Table 2. As a result, a higher Jsc of 8.70 mA/cm^2^ and a FF of 0.51 are simultaneously obtained. More interesting, all the parameters of the tandem cells have a low standard deviation, indicating that a high reproducibility of these tandem cells.

**Figure 6:**
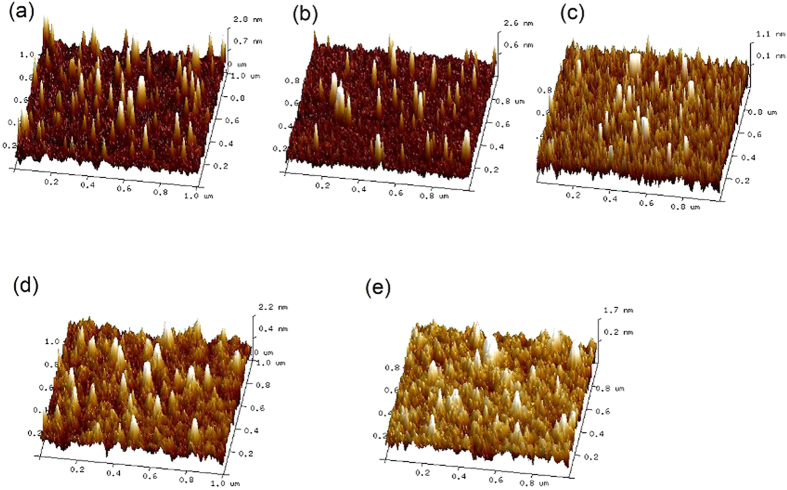
AFM images (1 μm × 1 μm) of (**a**) neat 5 nm thin film of MoO_3_ on Si substrate. (**b**–**e**) on Si substrate, neat 5 nm thin film of MoO_3_ coated with various SubPc thin film thickness (0.5, 1.0, 1.5 and 2 nm respectively). The root-mean-square (rms) roughness of the image from (**a**–**e**) were 0.478, 0.420, 0.341, 0.448 and 0.373 respectively.

To further understand the effects of the SubPc layer on the performance of the OPV cells, AFM images of SubPc with different thickness on 5 nm MoO_3_ were investigated, as presented in Fig. 6(a–e). The root-mean-square (RMS) roughness of pristine MoO_3_ layer is 0.478 nm and then changes to be 0.420, 0.341, 0.448 and 0.373 nm for 0.5, 1.0 nm, 1.5 nm and 2.0 nm SubPc on MoO_3_, respectively. This difference RMS roughness between the films indicates that 1.0 nm SubPc covers majority of MoO_3_ surface, while 0.5 nm SubPc has a lower coverage on MoO_3_ surface. The increase of RMS roughness with 1.5 nm SubPc should be attributed to the aggregation of the ultra thin SubPc layer, while it will form a uniform film when the thickness of SubPc increases to 2.0 nm due to the crystallization of SubPc, which resultes in decease of RMS roughness. In spite of this, the cell with 2 nm SubPc presents an increased series resistance, thus the FF and hence the PCE is decreased (See Fig. 2 and Table 1). The difference between the morphologies also corresponds to the EQE spectra of the cells with different thickness of SubPc, as shown in Fig. 3.

**Figure 7:**
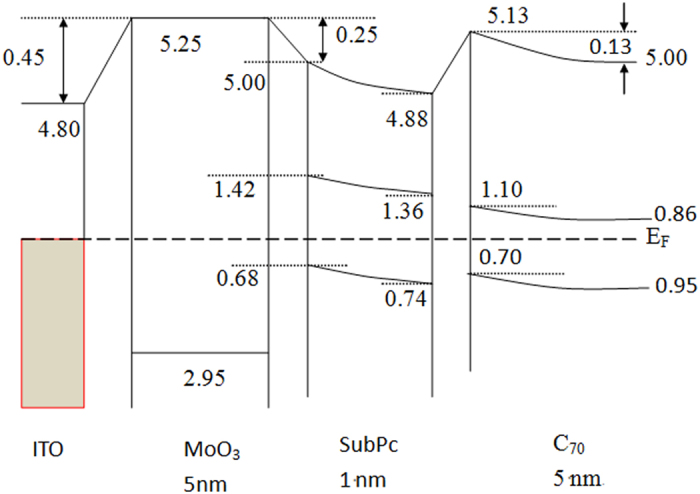
Schematic energy level alignment at the C_70_:5 wt%TAPC/SubPc/MoO_3_/ITO interfaces. The data for vacuum level (VL)and LUMO denote the values of energy level above the E_F_, while the data for HOMO denote those below the E_F_.

The energy level alignment of the cells plays an important role in determining the charge carrier extraction efficiency. Thus we investigate the energy level alignment at C_70_/SubPc/MoO_3_/ITO using UPS, as indicated in Fig. 7. As a result of the band bending in both TAPC and C_60_ layer deposited on MoO_3,_ a built-in field would be resided^23^. Here we only used pure C_70_ layer rather than the C_70_:5 wt% TAPC layer in the experiment. From Fig. 7, we note that there is an interface dipole of Δ_1_ = 0.45 eV at the MoO_3_/ITO interface, and this interface can not be changed by the subsequent deposition of SubPc^19^ as it only affects the surface state of MoO_3_. The vacuum level (VL), and valence band (VB) of MoO_3_ were measured to be 5.25, and 2.75 eV, respectively. A dipole of Δ_2_ = 0.25 eV is formed at the SubPc/MoO_3_ interface, inducing a behaviour of band bending in SubPc. The band-bending phenomenon in SubPc layer presents a drift electric field inside the organic material^17^,^19^. At the very interface of SubPc/MoO_3_, the VL, LUMO and HOMO of SubPc are 5.00, 1.42, and 0.68 eV, then they change to 4.88, 1.36, and 0.74 eV, respectively, with the increase of the thickness of SubPc. With the subsequent deposition of C_70_, the VL, LUMO, and HOMO of C_70_ are read as 5.13, 1.73, and 0.7 eV, respectively. A gradual energy level shift is observed with the further C_70_ deposition until to 5 nm, indicating exists a band bending region. So after C_70_:5 wt%TAPC mixed layer was deposited on SubPc/MoO_3_/ITO, a band bending in both SubPc and C_70_: TAPC BHJ towards MoO_3_ would take place^23^. The more detail analysis on these interfaces by UPS experiments would be discovered further. In view of these, the holes from C_70_:TAPC-BHJ can be easily transported to the anode via the bending HOMO level of SubPc due to the reduced holes extraction barrier.

**Figure 8:**
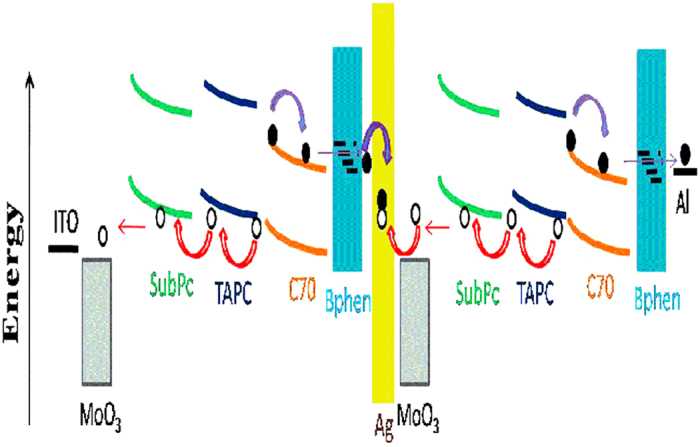
The schematic level alignment and band bending as well as the exciton dissociation, carrier transporting route and recombination in Ag ICL. White and black circles denote hole and electrons, bending red arrows show the carrier transport direction, bending green, blue and organic lines denote energy band bending of SubPc, TAPC and C_70_, respectively. The closer hole and electron lying in the yellow Ag ICL denote their recombination.

Thus the work mechanism of the tandem OPV cells can be explained as follows. Firstly, when the incident light was entered into such a tandem cell, the excitons are generated in the C_70_:5 wt%TAPC active layer of both the subcells due to the absorption of C_70_. The excitons of C_70_ can be quickly dissociated into free holes and electrons by the HOMO energy level offset between TAPC and C_70 _24 and aforementioned built-in field induced by both band bendings. Besides, the excitons of C_70_ can also be dissociated at the SubPc/C_70_ interface due to the HOMO energy level offset between these two materials, as shown in Fig. 7. Because the favorable energy level alignment at the MoO_3_/SubPc and SubPc/C_70_ interfaces can improve exciton dissociation and decrease the potential barrier for charge extraction, high Jsc and PCE were harvested. Secondly, the thinner total thickness of the two subcells in the tandem cell also possesses another advantage, i.e., the illumination light could enter the back subcell more easily. As a result, a balanced photocurrent would generate in the two subcells. Finally, after the excitons dissociate in the two subcells, the working processes and mechanisms are showed in Fig. 8, where the schematic level alignment, band bending, the exciton dissociation, carrier transporting route and recombination in Ag intermediate layer are clearly displayed. There are two built-in fields in the cell, one is arising from MoO_3_-SubPc Schottky-junction^13^,^17^ and the other one is from the offset between HOMO levels of C_70_ and TAPC. Under the driving of the built-in fileds in the front subcell, the photogenerated holes are transported to the anode via the HOMOs of TAPC and SubPc, while the electrons are transported to Ag intermediate layer through the LUMO of C_70_ and the defect states of Bphen. Similar situations will occur in the back subcell, but with the holes transported to Ag intermediate layer and electrons to Al cathode. Then recombination between holes and electrons takes place at Ag nanoparticles. Furthermore, it is also remarkable that the very thin SubPc interlayer not only induces band bending of C_70_:5 wt%TAPC layer but also prevents exciton quenching by MoO_3_. Exciton quenching at the MoO_3_/organic interface plays an important role in determining the PCE of the OPV cells, especially in the cells where the BHJ active layer with a relatively high concentration of C_70_ is directly contacted with MoO_3_^25^. In a word, the introduction of a SubPc organic layer in our tandem cell does improve the performance of the tandem cells and a more detailed working mechanism is still under research.

## Conclusion

We have demonstrated an efficient tandem OPV cell with identical subcells based on C_70_:5 wt%TAPC. By inserting an ultrathin SubPc layer, a PCE as high as 7.27% was demonstrated. The response of the cell primary contributed from the absorption of C_70_. The high efficiency was attributed to the high absorption efficiency of C_70_ and improved holes extraction efficiency at the anode due to the band bending occurs at the MoO_3_/SubPc and SubPc/C_70_:5 wt%TAPC interfaces. These findings indicate that high PCE tandem OPV cells with identical subcells can be constructed by precise device design, and this work provides a new strategy to fabricate high performance tandem OPV cells.

## Methods

### Fabrication of small molecule materials based organic photovoltaic cells

The organic materials used for all the OPV cells were procured commercially and were used without further sublimation. The ITO-coated glass substrates with a sheet resistance of 15 Ω/sq were routinely cleaned in a series of solvents and then treated by ultraviolet-ozone in a chamber for 15 min. Organics and metal layers were sequentially deposited onto the clean patterned ITO substrates by thermal evaporation in a vacuum chamber at a pressure of 4 × 10^−4^ Pa without a vacuum break. The area of the devices patterned by the shadow mask was 0.1 cm^2^. The deposition rates were monitored using quartz oscillating crystals and kept to be 0.5 Å/s for organic layer and 5 Å/s for the Al cathode.

### Device measurement and characterization

Absorption spectra of the organic films on quartz substrates were measured with a Shimadzu UV-3101PC spectrophotometer. The current density–voltage (J–V) characteristics of the OPV cells were measured using a computer-controlled Keithley 2611 source meter under AM 1.5 G illumination from a calibrated Solar with an irradiation intensity of 100 mW·cm^−2^. The external quantum efficiency (EQE) measurements were performed with a lock-in amplifier at a chopping frequency of 20 Hz during illumination with monochromatic light from a xenon lamp and their intensities are calibrated with a Si-photodiode. The surface topographies were imaged with a Bruker MultiMode 8 atomic force microscope (AFM) in tapping mode. The UPS experiments were performed using a VG ESCA Lab system equipped with a He I (21.22 eV) gas discharge lamp. The ultrahigh vacuum (UHV) system consists of a spectrometer chamber and an evaporation chamber. The base pressures of the spectrometer chamber and the evaporation chamber are typically 1.1 × 10^−8^ Pa and 6.7 × 10^−6^, respectively. We recorded the UPS spectra with the samples biased at −4.0 V to observe the true low energy secondary cut-off. The UV light spot size on samples was about 1 mm in diameter. The instrumental resolution for UPS measurements was chosen to be 10 meV. All the measurements were carried out at room temperature.

## Additional Information

**How to cite this article**: Gao, Y. *et al*. Highly efficient organic tandem solar cell with a SubPc interlayer based on TAPC:C_70_ bulk heterojunction. *Sci. Rep.* **6**, 23916; doi: 10.1038/srep23916 (2016).

## Supplementary Material

Supplementary Information

## Figures and Tables

**Table 1:** PV parameters and series resistance (Rs) of single cell with different thickness of SubPc.

Thickness (nm)	Jsc(mA/cm^2^)	Voc(V)	FF	PCE(%)	R_s_ (ohm.cm^2^)
0.0	8.94 ± 0.07	0.85 ± 0.01	0.51 ± 0.01	3.88 ± 0.12	7.73
0.5	10.40 ± 0.02	0.86 ± 0.01	0.52 ± 0.02	4.63 ± 0.27	5.79
1.0	10.98 ± 0.01	0.85 ± 0.01	0.53 ± 0.01	4.95 ± 0.06	2.69
1.5	9.55 ± 0.19	0.84 ± 0.01	0.52 ± 0.01	4.23 ± 0.01	3.54
2.0	10.46 ± 0.21	0.79 ± 0.01	0.45 ± 0.01	3.75 ± 0.18	9.67

**Table 2:** The PV parameters of a series of tandem cells with different thickness of active layer of C_70_:5 wt%TAPC, respectively.

Thickness (x, y nm)	Jsc(mA/cm^2^)	Voc(V)	FF	PCE(%)	R_s_ (ohm.cm^2^)
(24.5, 49)	6.19 ± 0.16	1.57 ± 0.01	0.56 ± 0.01	5.42 ± 0.16	11.19
(25, 49)	6.27 ± 0.15	1.58 ± 0.04	0.57 ± 0.03	5.63 ± 0.37	13.41
(25, 54)	7.57 ± 0.35	1.58 ± 0.01	0.48 ± 0.01	5.79 ± 0.33	42.54
(28, 54)	6.80 ± 0.17	1.59 ± 0.01	0.57 ± 0.03	6.14 ± 0.16	19.00
(28, 59)	6.69 ± 0.19	1.50 ± 0.01	0.56 ± 0.01	5.63 ± 0.19	9.30
(28, 64)	7.09 ± 0.07	1.56 ± 0.01	0.41 ± 0.01	4.80 ± 0.23	15.40
(28, 74)	5.47 ± 0.34	1.51 ± 0.03	0.39 ± 0.05	3.26 ± 0.43	43.36
(33, 54)	8.70 ± 0.23	1.63 ± 0.01	0.51 ± 0.01	7.27 ± 0.18	42.69
Single cell	10.98 ± 0.01	0.85 ± 0.01	0.53 ± 0.01	4.95 ± 0.06	2.69
