# Supplementary material for: Highly efficient organic tandem solar cell with a SubPc interlayer based on TAPC:C70 bulk heterojunction
Source: Sci Rep. 2016 Apr 1;6:23916. doi: 10.1038/srep23916 (PMC4817034; doi:10.1038/srep23916)
Supplement: Supplementary Information [file srep23916-s1.doc]

Supporting Information

**Highly efficient organic tandem solar cell with a SubPc interlayer based on TAPC:C70 bulk heterojunction**

*Yuan Gao,^1,2^ Fangming Jin,^1,2^ Wenlian Li,^1*^ Zisheng Su,^1*^ Bei Chu,^1^ Junbo Wang,^1^ Haifeng Zhao,^1^ Hairuo Wu,^1,2^ Chengyuan Liu,^1,2^ Fuhua Hou^1,2^ Tong Lin,^1,2^ and Qiaogang Song ^1,2^*

^1^State Key Laboratory of Luminescence and Applications, Changchun Institute of Optics, Fine Mechanics, and Physics, Chinese Academy of Sciences, Changchun 130033, People’s Republic of China

^2^University of Chinese Academy of Sciences, Beijing 100039, People’s Republic of China

**Corresponding Authors**

*E-mail: [wllioel@aliyun.com](mailto:wllioel@aliyun.com) and [suzs@ciomp.ac.cn](mailto:suzs@ciomp.ac.cn)

Tel.: +86-431-86176345

Fax: +86-431-86176345.


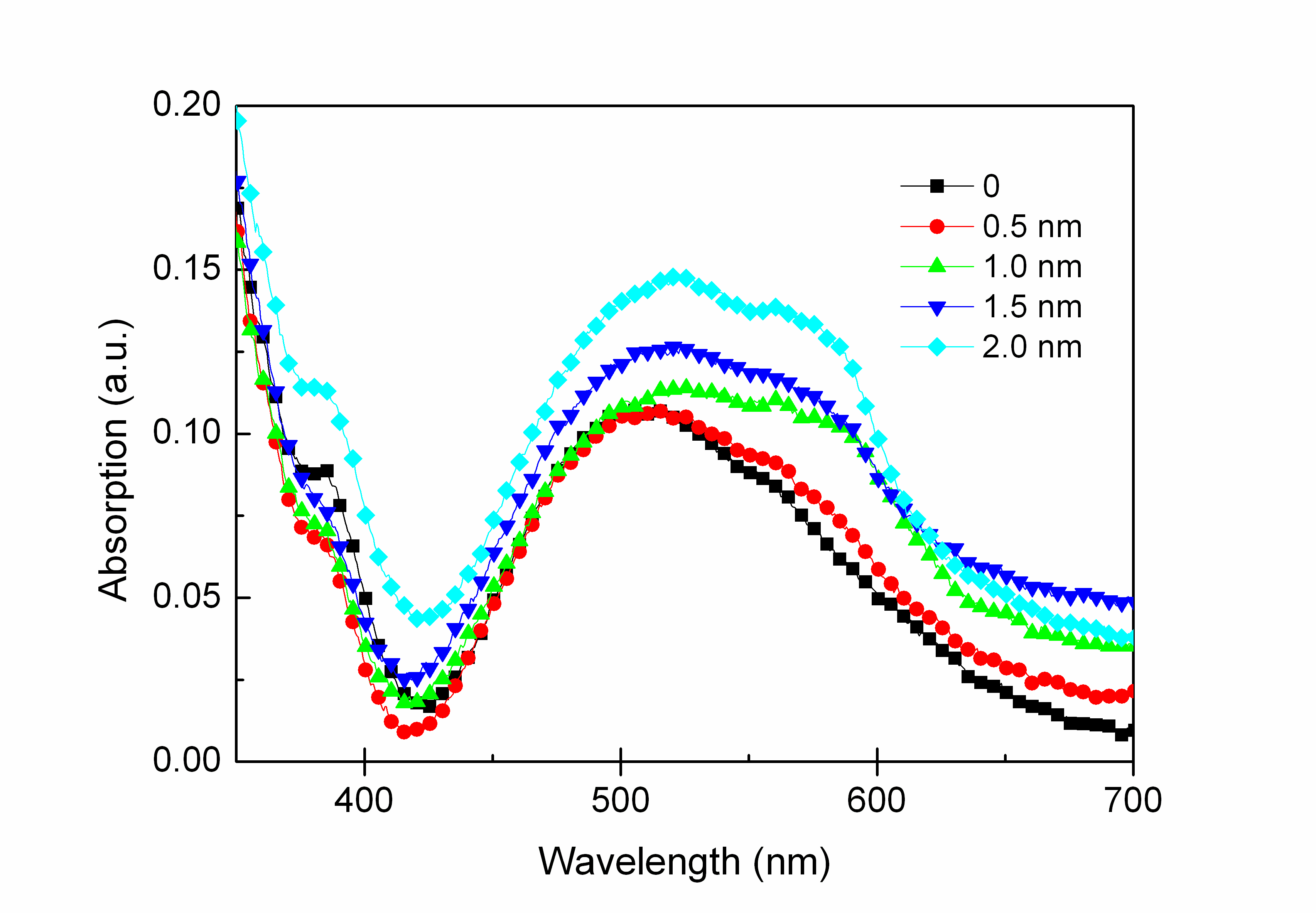


**Figure S1.** Absorption spectra of the SubPc/C_70_:TAPC films with different thickness of SubPc.

**
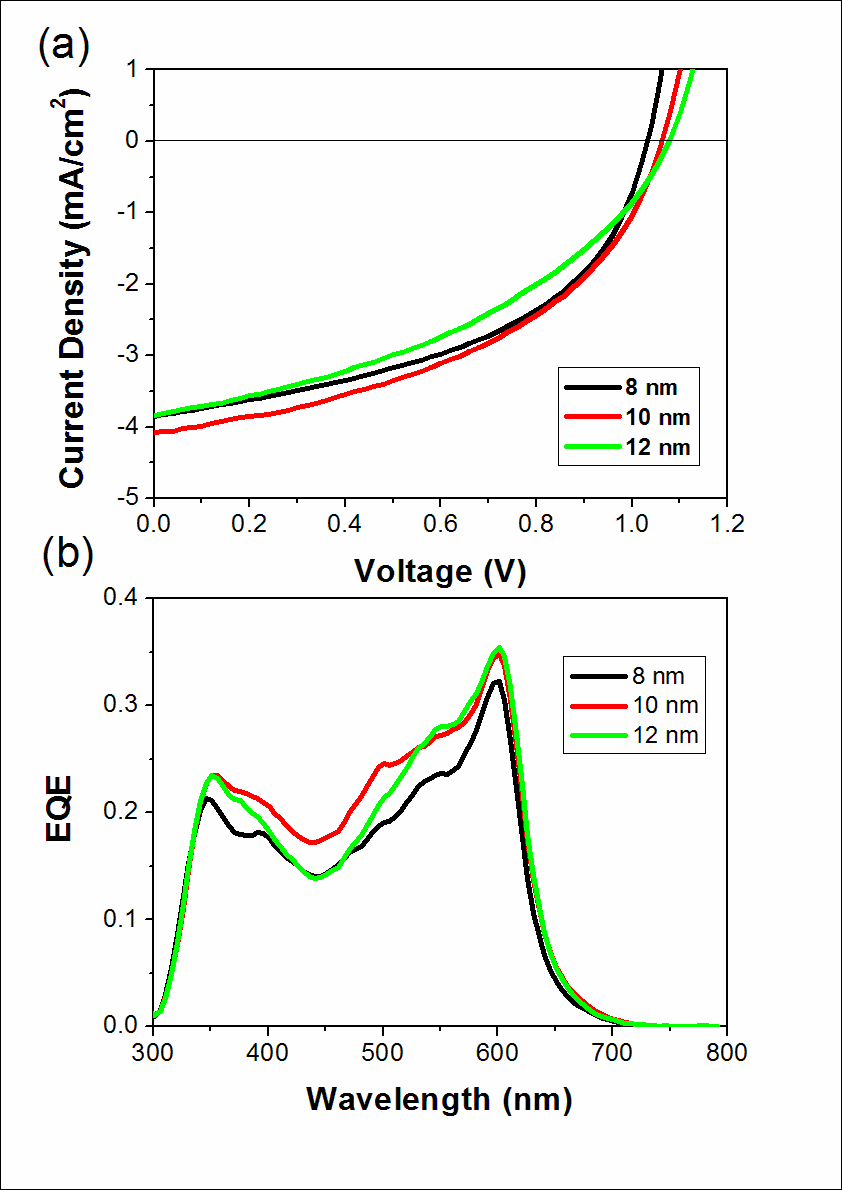
**

**Figure S2.** (a) J-V curves and (b) EQE of the devices ITO/MoO_3_ (5 nm)/SubPc/C_70_ (35 nm) /Bphen (8 nm)/Al with different thickness of SubPc.


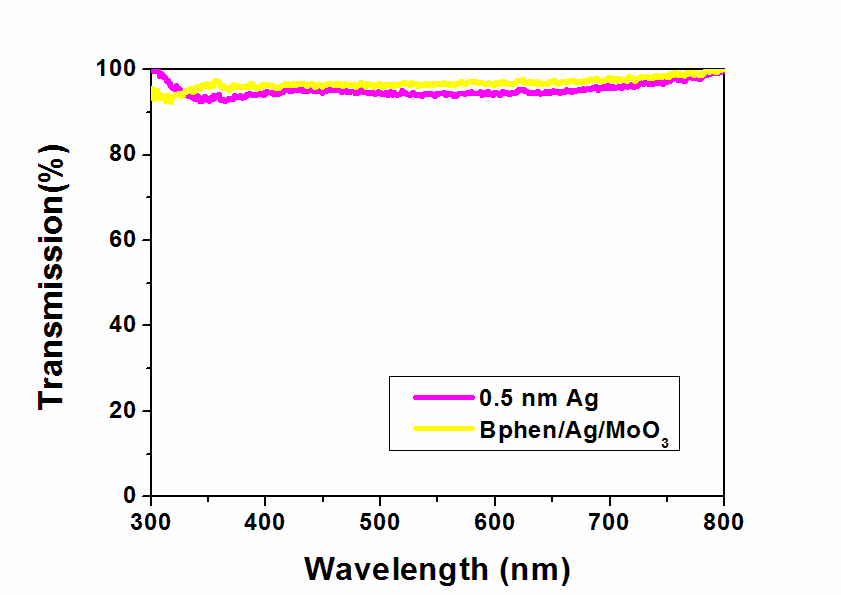


**Figure S3.** Transmittance spectrum of Ag (0.5 nm) and Bphen (2 nm)/Ag (0.5 nm) /MoO_3_ (5 nm) films.
